# The Good and Bad of Nrf2: An Update in Cancer and New Perspectives in COVID-19

**DOI:** 10.3390/ijms22157963

**Published:** 2021-07-26

**Authors:** Sonia Emanuele, Adriana Celesia, Antonella D’Anneo, Marianna Lauricella, Daniela Carlisi, Anna De Blasio, Michela Giuliano

**Affiliations:** 1 Department of Biomedicine, Neurosciences and Advanced Diagnostics (BIND), University of Palermo, Via del Vespro 129, 90127 Palermo, Italy; adriana.celesia@unipa.it (A.C.); marianna.lauricella@unipa.it (M.L.); daniela.carlisi@unipa.it (D.C.); 2 Department of Biological, Chemical and Pharmaceutical Sciences and Technologies (STEBICEF), Biochemistry Building, University of Palermo, Via del Vespro 129, 90127 Palermo, Italy; antonella.danneo@unipa.it (A.D.); anna.deblasio@unipa.it (A.D.B.); michela.giuliano@unipa.it (M.G.)

**Keywords:** Nrf2, oxidative stress, cell death, cancer, COVID-19, NF-κB, inflammation

## Abstract

Nuclear factor erythroid 2-related factor 2 (Nrf2) is a well-known transcription factor best recognised as one of the main regulators of the oxidative stress response. Beyond playing a crucial role in cell defence by transactivating cytoprotective genes encoding antioxidant and detoxifying enzymes, Nrf2 is also implicated in a wide network regulating anti-inflammatory response and metabolic reprogramming. Such a broad spectrum of actions renders the factor a key regulator of cell fate and a strategic player in the control of cell transformation and response to viral infections. The Nrf2 protective roles in normal cells account for its anti-tumour and anti-viral functions. However, Nrf2 overstimulation often occurs in tumour cells and a complex correlation of Nrf2 with cancer initiation and progression has been widely described. Therefore, if on one hand, Nrf2 has a dual role in cancer, on the other hand, the factor seems to display a univocal function in preventing inflammation and cytokine storm that occur under viral infections, specifically in coronavirus disease 19 (COVID-19). In such a variegate context, the present review aims to dissect the roles of Nrf2 in both cancer and COVID-19, two widespread diseases that represent a cause of major concern today. In particular, the review describes the molecular aspects of Nrf2 signalling in both pathological situations and the most recent findings about the advantages of Nrf2 inhibition or activation as possible strategies for cancer and COVID-19 treatment respectively.

## 1. Introduction

Redox signalling has emerged as a fine mechanism that controls cell fate and is involved in carcinogenesis and tumour progression [1] as well as inflammation and response to viral infections [2].

Nrf2 is a well-known oxidative response transcription factor that exerts cytoprotective effects following pro-oxidant stimuli due to electrophilic or oxidative stress. Together with its negative regulator Keap1, Nrf2 represents a main player of the defence response to both extrinsic and intrinsic forms of cellular stress, giving rise to what is best known as Nrf2/Keap1 signalling pathway. Under normal conditions, when cell stress is low or moderate, Keap1 binds to Nrf2 and promotes its ubiquitination and proteasome-mediated degradation. Nrf2 levels are thus kept low in the cell due to the fine balance with Keap1 activity.

Keap1 is a sensor protein, which contains cysteine residues that can be modified by reactive oxygen species (ROS). Under the condition of oxidative stress, Keap1 undergoes a conformational change that decreases affinity to Nrf2 [3]. Consequently, Nrf2 can be released and translocate to the nucleus, where it stimulates the transcription of antioxidant and pro-survival genes.

Therefore, under healthy conditions, Nrf2 serves a protective function against tumourigenesis and tumour progression by counteracting ROS-induced toxicity and genotoxic stress. However, persistent stimulation of Nrf2 response can promote cell survival and create an optimal environment for cell proliferation and consequent tumour transformation. In particular, Nrf2 has been shown to protect tumour cells from oxidative stress, being the redox status altered in tumour cells due to high ROS levels. It has been also correlated with chemoresistance as well as metabolic reprogramming to anabolic routes favouring tumour growth and expansion. In this perspective, Nrf2 assumes a dual role in cancer that has been recognised by other authors [4,5].

It is clear that tumour cells display peculiar features such as excessive stress due to ROS imbalance and this may cause Nrf2 hyperactivation or Keap1 downregulation, thereby shifting the balance toward antioxidant and pro-survival response. Another factor that may contribute to Nrf2 persistent activation in tumour cells is the activation of p62, a multifunctional protein involved in selective autophagy and often overexpressed in tumours [6]. P62 in a phosphorylated form can interact with Keap1 at the same binding site for Nrf2 and thus competitively inhibits the Keap1/Nrf2 interaction resulting in Nrf2 stabilization and translocation to the nucleus. Nrf2, in turn, can stimulate p62 gene expression thus amplifying a pro-survival circuit that can favour tumourigenesis. Intriguingly, p62 has also a Janus behaviour as we previously described [6].

The aforementioned considerations imply that Nrf2 may be considered a target in some situations, thus supporting a possible use of specific inhibitors, or rather a defensive weapon to activate, thus stimulating the interest towards its activators.

Activation of Nrf2 has been recently recognised as a possible tool to counteract inflammation and cytokine storms that occur under viral infections. In this context, some authors have highlighted the potential of Nrf2 activators for the treatment of coronavirus disease 19 (COVID-19) [7,8]. However, the benefits of stimulating Nrf2 mediated response against the viral disease need a better evaluation and further investigation.

In such a variegated scenario, where two widespread diseases, cancer and COVID-19, continue to alarm, we decided to provide a critical focus on the molecular aspects dissecting Nrf2 dual role and summarize in an updated review the most recent findings about the advantages of Nrf2 activation or inhibition in both diseases.

## 2. Nrf2 Structure and Function

### 2.1. Nrf2 Structure and Post-Translational Modifications

**Figure 1:**
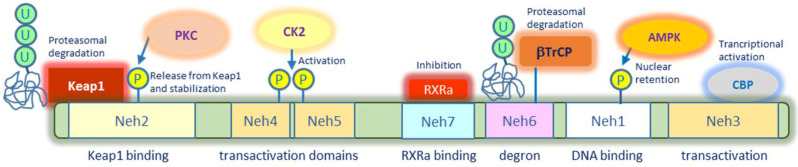
Molecular structure of Nrf2 and post-translational modifications. Seven highly conserved Nrf2-ECH homology (Neh) domains are described together with their main interactors and modifications. Neh1 is the DNA binding domain at specific antioxidant response elements (ARE) and is susceptible to phosphorylation by AMP kinase that causes Nrf2 nuclear retention. Neh2 is the Keap1 binding domain and is subjected to ubiquitination and consequent proteasomal degradation. Neh3 is the transactivation domain and recruits co-activators such as CBP. Neh4 and Neh5 also contribute to transactivation and phosphorylation within these domains promotes Nrf2 transcriptional activity. Neh6 is an alternative degradation domain that recognizes the ubiquitin E3 ligase β-TrCp. The Neh7 domain binds to retinoid X receptor α (RXRα) that suppresses Nrf2 transcriptional activity.

Nrf2 is encoded by the NFE2L2 gene, which belongs to the family of transcription factors cap and collar (CNC) containing a basic leucine zipper DNA binding domain at the C terminus. Concerning the structure, Nrf2 displays seven highly conserved Neh (Nrf2-ECH homology) domains, from Neh1 to Neh7 (Figure 1). In particular, Neh1 is responsible for DNA binding at specific antioxidant response elements (ARE), which are present in promoter/enhancer regions of several genes encoding antioxidant and detoxifying enzymes as well as pro-survival factors [9]. This domain can also permit Nrf2 heterodimerization with sMafs (small musculo-aponeurotic fibrosarcoma) proteins [10,11]. Binding to these proteins promotes gene transactivation and retains Nrf2 to the nucleus since they mask the nuclear export sequence (NES) present within Neh1 domain [10]. Nuclear export of Nrf2 may be also prevented by phosphorylation in Ser550 present in this domain promoted by AMP kinase, a well-known regulatory key component of energy deficient metabolic response in cells [12].

The Neh2 domain, which is located at the N terminus region, contains DLG and ETGE motifs that are critical for Keap1 binding and consequent Nrf2 degradation. Lysine residues present in this domain account for Keap1-mediated Nrf2 ubiquitination to target the factor to 26S proteasome. Interestingly, Nrf2 phosphorylation in this domain may affect Keap1 binding and thus stabilise Nrf2 impeding proteasomal degradation. Recent studies suggest that protein kinase C (PKC) phosphorylates Nrf2 in the Neh2 domain to favour the release from Keap1 and consequent Nrf2 stabilization [13].

The Neh3 domain, located at the C terminus, together with Neh4 and Neh5 domains, is responsible for transcriptional activation. All the three transactivation domains are able to recruit co-activators and histone acetyltransferases including CREB binding protein (CBP) and Brahma-related gene1 (BRG1) to promote gene expression [14]. Post-translational modifications within Neh4 and Neh5 domains include Casein Kinase 2 (CK2)-mediated phosphorylation, which seems to be necessary for Nrf2 activation [15].

The Neh6 domain is fundamental for Nrf2 alternative degradation since it is rich in serine residues that are subjected to phosphorylation by glycogen synthase kinase-3 (GSK-3). Specifically, upon GSK-3 phosphorylation Nrf2 can bind to β-TrCP, another ubiquitin ligase that is independent of the redox status of the cell, but sensitive to cell cycle regulation and nutrient sensing [16].

The Neh7 domain has been shown to bind to retinoid X receptor α (RXRα) that negatively modulates Nrf2, thus suppressing its transcriptional activity [17].

Nrf2 can also be regulated by other different kinases including mitogen activated protein kinases (MAP kinases) such as p38, JNK and ERK with diversified effects [18]. Another kinase that can phosphorylate Nrf2 is RNA-like endoplasmic reticulum kinase (PERK), which triggers the unfolded protein response (UPR) following endoplasmic reticulum (ER) stress. Phosphorylated PERK activates Nrf2 independently of oxidative stress but following UPR, and stabilises the factor due to destabilization of its interaction with Keap1. For a complete description of Nrf2 phosphorylation patterns, the interesting review of Liu et al. deserves attention [12].

### 2.2. Nrf2-Mediated Signalling Pathways and Regulatory Network

#### 2.2.1. Classical Nrf2 Transcriptional Targets

**Figure 2:**
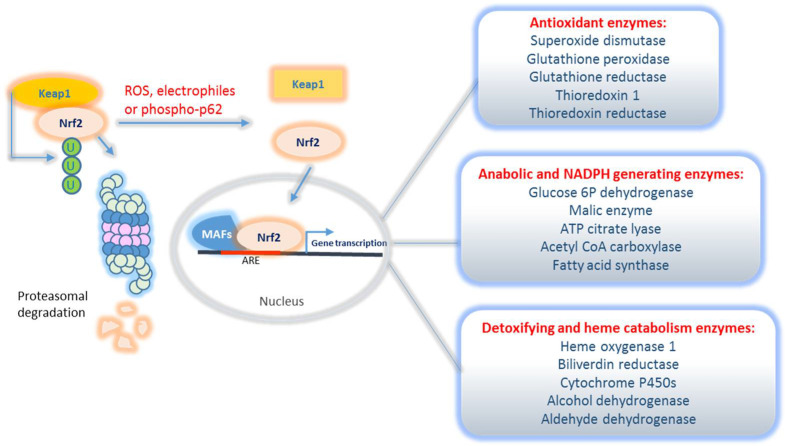
Molecular targets and pathways regulated by Nrf2. Under normal conditions, Keap1 binds to Nrf2 and promotes its ubiquitination and proteasome-mediated degradation. Under electrophilic or oxidative stress conditions, Keap1 undergoes a conformational change that decreases affinity to Nrf2. Consequently, Nrf2 is released and translocates to the nucleus, where it stimulates the transcription of a battery of genes including antioxidant, detoxifying and anabolic ones.

It is clear that Nrf2, once released from Keap1 binding, is able to maintain the redox homeostasis and to promote cell survival by activating the transcription of its multiple cytoprotective target genes containing antioxidant response elements (ARE) in their promoters (Figure 2). Among the classical Nrf2 transcriptional targets, not only a well-known battery of antioxidant enzymes exists, such as superoxide dismutase (SOD) and glutathione peroxidase (GPX) to scavenge ROS, but also two other big classes of genes involved in detoxification and anabolic reprogramming, respectively. Briefly, the first class of enzymes is involved in the oxidation of xenobiotics or drugs, conjugation reactions, waste disposal, thus cooperating to serve a cytoprotective function. This class of enzymes includes heme oxygenase and cytochrome P450, as the main component. The second class includes genes encoding enzymes involved in NADPH generation and anabolic reactions. NADPH is an essential co-factor for both xenobiotic conjugation and antioxidant enzymes as well as a key element to switch cell metabolism toward the anabolic phase. Nrf2-regulated metabolic pathways indeed include the pentose phosphate pathway and stimulation of enzymes that cooperate with fatty acid biosynthesis [19]. The specific description of Nrf2 transcriptional targets is beyond the scope of this review but the papers by Liu et al. and Ibrahim et al. can provide more insights [20,21].

#### 2.2.2. Interplay Nrf2/NF-κB and CBP

Dissecting the fine regulation of Nrf2-mediated transcriptional activity and its interactive nexus at the nuclear level is an arduous task, especially considering the complicated nuclear network that includes epigenetic regulation. However, among the best-characterised nuclear factors interplaying with Nrf2, NF-κB and CBP deserve particular attention.

NF-κB represents a family of transcription factors that are involved in inflammatory and pro-survival responses linking chronic inflammation to both cancer and viral infections.

**Figure 3:**
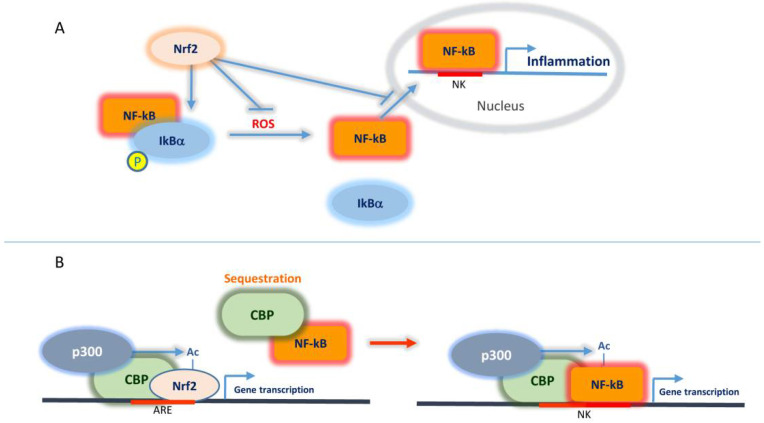
Mutual regulation between Nrf2 and NF-κB. (**A**) Nrf2 inhibits NF-κB at different levels: (i) it contributes to IκB-α stabilization and consequent NF-κB retention to the cytoplasm and subsequent degradation, (ii) it attenuates ROS levels thus affecting one major NF-κB activating stimulus, (iii) it interferes with NF-κB nuclear translocation. (**B**) Nrf2 interacts and cooperates with CBP/p300 at the nuclear level to promote gene transcription. NF-κB can compete with Nrf2 for CBP binding thus sequestering the factor and recruiting it to specific NF-κB response elements.

In a canonical pathway and under normal cell conditions, NF-κB remains inactive since it is sequestered in the cytoplasm by its inhibitory factors, including IkB proteins. Under specific stimuli including cell stress, IkB proteins become phosphorylated by the IkB kinase complex causing their ubiquitination and proteasomal degradation. Consequently, NF-κB is released and translocates to the nucleus where it transactivates multiple target genes strongly promoting inflammation [22,23]. Hence, while the same stimulus of oxidative stress may activate both Nrf2 and NF-κB, the former represents a cellular protective factor and the latter stimulates cytokine-mediated inflammation and consequent cellular injury. Therefore, it is not surprising that Nrf2 negatively modulates the NF-κB-mediated signalling. As a first level of negative regulation, Nrf2, through its antioxidant response, reduces ROS levels in the cells thus attenuating NF-κB activating stimulus. Moreover, Nrf2 has been shown to contribute to IkB-α stabilization and consequent NF-κB retention to the cytoplasm and subsequent degradation [24,25]. A further level of NF-κB inhibition by Nrf2 occurs at the nuclear level and involves CBP protein. This latter, which interacts with the histone acetylase p300 favouring epigenetic positive control of gene transcription, has been shown to act as a transcriptional co-activator of Nrf2 (Figure 3) [26]. Beyond histone tails, the p300/CBP complex can also acetylate lysine residues of other proteins among which there are both Nrf2 and the p65 subunit of NF-κB [27]. Acetylation of the Neh1 domain of Nrf2 by p300/CBP favours Nrf2 DNA binding and consequent transcription of Nrf2 target genes. On the other hand, p65-NF-κB has been shown to compete for CBP binding thus limiting CBP interaction with Nrf2 and thus contributing to Nrf2 transcriptional suppression. Moreover, evidence has been provided that p65 can interact with Keap1 promoting Nrf2 degradation [28]. Considering that Keap1 can also direct Nrf2 nuclear export [29], it remains to be elucidated whether p65/Keap1 interaction and subsequent Nrf2 inactivation occurs in the nucleus or in the cytoplasm.

Overall, it is clear that a mutual negative regulation occurs between Nrf2 and NF-κB, which will be further considered in the next paragraphs in the context of cancer and inflammatory response promoted by COVID-19 viral infection.

#### 2.2.3. Nrf2 Relationship with p53 and p21

**Figure 4:**
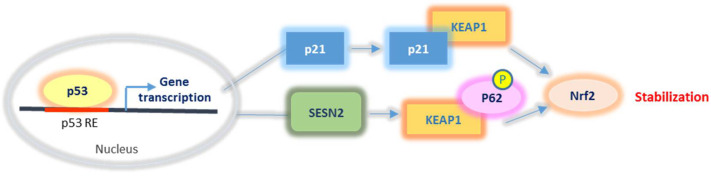
The effects of p53 on Nrf2. P53 contributes to Nrf2 stabilization by promoting the expression of p21/WAF1, which sequesters Keap1, thus promoting Nrf2 release and activation. P53 also promotes sestrin 2 (SESN2) gene transcription. SESN2 stimulates Keap1 degradation via the selective autophagy mediator p62 thus contributing to Nrf2 stabilization.

P53 represents another key factor that can be subjected to redox regulation and interplays with Nrf2. Beyond its well-characterised role in cell cycle arrest and apoptosis, p53 can also induce the transactivation of antioxidant or pro-oxidant genes depending on its cellular level. Intriguingly, low levels of p53 seem to promote the expression of genes with antioxidant functions, whereas higher p53 levels, resulting from considerable stress conditions, induce pro-oxidant and pro-apoptotic target genes [30]. Among early response p53 targets, Sestrins 1 and 2 (SESN1/2) and p21/WAF1 have been shown to promote Nrf2 stabilization and activation (Figure 4). In particular, SESN1/2 can exert control of the cellular redox status by promoting Keap1 degradation via p62 interaction and consequent Nrf2 release and activation [31]. In addition, p21 has been shown to compete with Keap1 for Nrf2 binding, thus contributing to inhibit Nrf2 sequestration and degradation. Notably, in its turn, Nrf2 can also promote the transcription of SESN2 [32], p21 [33], and p53 negative regulator MDM2. While SESN2 and p21 expression by Nrf2 represents potentiating feedback, the regulation of MDM2 expression contributes to favouring p53 turnover thus restoring p53 low levels. In this context, it is clear that low levels of p53 may exert a positive effect on Nrf2 activation, while conditions that promote considerable p53 increase can conversely compromise Nrf2 function [34].

It is interesting to note that mutant p53 can contribute to maintaining antioxidant levels in tumour cells by activating Nrf2. In this regard, evidence has been provided that a mutated p53 form can sustain Nrf2-mediated antioxidant response to allow cancer cell adaptation to the tumour-typical increased ROS levels [35] and to confer chemoresistance [36].

#### 2.2.4. Nrf2 Interaction with Kinases and Acetylases

As previously mentioned, Nrf2 can be finely regulated by post-translational modifications including phosphorylation and acetylation. Among the kinase-mediated signalling that influences Nrf2 activity, it is worth mentioning the phosphoinositide-3-kinase (PI3K) pathway, the AMP kinase-dependent energy deficient metabolic response and the PERK-induced unfolded protein response (UPR). Concerning PI3K, it is well known that this signalling is activated following insulin or insulin-like growth factor stimulation and culminates in protein kinase B (PKB), also named AKT, activation. The control of Nrf2 by PI3K is exerted at different levels. First, AKT overexpression has been shown to promote Nrf2 activation [37]. Second, PI3K inhibits glycogen synthase Kinase-3β (GSK-3β), which phosphorylates Nrf2 in Neh6 degron domain thus promoting βTRCP-mediated Nrf2 degradation. In this way, PI3K contributes to Nrf2 stabilization. Third, the tumour suppressor phosphatase and tensin homolog (PTEN), which is known to counteract PI3K signalling has also been demonstrated to inhibit Nrf2. Interestingly, in the liver of PTEN-deficient mice, Nrf2 activation and nuclear localization was observed [38].

AMP kinase is activated in response to energy deprivation when cellular ATP levels decrease whereas AMP levels increase. Beyond the well-known function of AMP kinase in stimulating catabolic pathways, evidence has been provided that the enzyme also contributes to Nrf2 phosphorylation and nuclear retention with consequent increased transcriptional activity [39]. Moreover, AMP kinase can also promote Nrf2 stabilization since it is capable of inhibiting GSK-3β, thus impeding βTRCP-mediated Nrf2 degradation [40].

Nutrient deprivation can also account for stimulation of the UPR. Cells indeed activate this pathway also in response to defects in protein folding that may occur under stress conditions, including starvation. Protein kinase RNA-like endoplasmic reticulum kinase (PERK) represents a key kinase triggering UPR response starting from the endoplasmic reticulum [41,42]. Evidence has been provided that PERK-dependent Nrf2 phosphorylation promotes its dissociation from Keap1 and consequent Nrf2-mediated signalling activation [43].

In addition to phosphorylation, lysine acetylation can also modulate Nrf2 function and influence its stability [44]. More specifically, P300 acetylase has been shown to interact with and to acetylate Nrf2 [45].

The implication of Nrf2 in response to cell stress, cell metabolism and cell fate determination open a new perspective to understand its role in both cancer development and cellular response to viral infections, two complex pathological conditions that deserve particular attention nowadays.

## 3. The Dual Role of Nrf2 in Cancer

The broad-spectrum action of Nrf2 and interactive network makes it a key factor in regulating the balance between cell death and survival, which is decisive in cell transformation leading to cancer development. Accumulating evidence indicates a contradictory role of Nrf2 in cancer. Being a factor that guarantees defence mechanisms and cell survival, in normal cells Nrf2 physiologically protects against tumourigenesis and tumour progression by attenuating cell stress and consequent genotoxicity. However, Nrf2-mediated defence response can also generate an optimal environment for cell proliferation, thus promoting cell transformation. In particular, it contributes to protect tumour cells from oxidative stress and favour metabolic reprogramming to anabolic pathways that sustain tumour development. Although other authors have already highlighted the double role of Nrf2 in cancer [4], the present paragraph aims to critically revise and discuss the most recent literature in this regard and to provide an updated view of the molecular mechanisms underlying the Janus role of Nrf2 in cancer.

### 3.1. The Anti-Tumour Role of Nrf2

Under healthy conditions, the Nrf2 general function of maintaining cellular redox homeostasis contributes to regulating cell growth and preventing tumourigenesis. For instance, evidence has been provided that Nrf2-deficient mice are extremely susceptible to redox imbalance and easily develop tumours due to increased DNA damage and consequent transformation [46]. Nrf2 role in protecting cells from oxidative stress also prevents the typical increase in ROS levels that often characterises tumour cells. Moreover, it is well known that tumourigenesis is a phenomenon that is strictly associated with inflammation and that Nrf2, exerting an anti-inflammatory function, contributes to counteracting inflammation-induced tumour transformation. More specifically, inhibition of NF-κB by Nrf2, as previously discussed, reduces the expression of pro-inflammatory cytokines such as tumour necrosis factor-α (TNFα), interleukines IL1, IL6, IL8 as well as the NF-κB-mediated processes including epithelial to mesenchymal transition [47] and tumour vascularization via upregulation of vascular endothelial growth factor (VEGF) and its receptors [48].

In addition, through the antioxidant response, Nrf2 induces the expression of thioreodoxin, an antioxidant enzyme that links oxidative stress to inflammasome modulation [49] and reduces inflammation in mice models [50]. Reduced ROS levels by the Nrf2 pathway have been also shown to decrease inflammation in hepatocarcinoma and in non-alcoholic fatty liver disease rat models, since Nrf2 silencing or inhibition of its targets superoxide dismutase and glutathione peroxidase abolished NF-κB inhibition and reduction in inflammatory cytokine secretion induced by the flavonoid hesperetin [51].

Similarly, Zhang et al. recently showed that the natural compound pterostilbene protects lipopolysaccharide-induced acute lung injury in mice through Nrf2-mediated inhibition of Cyclooxygenase-2 (COX-2), nitric oxide synthase (iNOS), TNF-α, IL-6 and IL-1β thus attenuating inflammatory response and oxidative stress [52].

Among the transcriptional targets of Nrf2, heme-oxygenase 1 (HO-1) not only takes part in heme catabolism but also exerts diversified roles in cancer. In the context of cancer prevention, HO-1 has been shown to be cytoprotective and to potently prevent inflammation. For instance, a very recent paper by Ebrahimpour et al. demonstrates that esomeprazole, a well-known inhibitor of the proton pump, attenuates inflammatory and fibrotic response in lung cells through the Nrf2/HO-1 pathway [53]. In accordance, other authors have shown that induction of HO-1 in macrophages strongly promotes anti-inflammatory activity [54]. Moreover, HO-1 knockout experiments have revealed a preventive role of the Nrf2/HO-1 axis in inflammation. In particular, HO-1 depletion caused systemic inflammation and vascular endothelial injury in both mice and humans [55].

However, it is important to specify that there are also conflicting data for HO-1 function in promoting anti-tumour processes since many other studies support a pro-tumour activity of HO-1 rendering this enzyme a double-faced molecule in cancer as well as Nrf2.

HO-1 is predominantly considered a pro-tumour enzyme since it is overexpressed in a wide range of cancers and has been shown to favour tumour progression by different mechanisms [56]. This aspect will be considered in the next subparagraph.

Nevertheless, based on the aforementioned studies, it is clear that the activation of Nrf2/HO-1-mediated signalling pathway plays a certain role in attenuating chronic inflammation, which is associated with cancer.

In this regard, a recent paper by Hemmati et al. highlights the importance of HO-1 in gastrointestinal cancers as a chemo-preventive agent, when acting as immunomodulator and heme detoxifying enzyme, and pro-tumour agent, when upregulated in colon cancer cells. The same authors recognise the Nrf2/HO-1 axis as a key protector of the intestinal mucosa under physiological conditions and stimulation by the microbiota given the important role in the disposal of heme, which is extremely toxic and carcinogenic for the colon [57].

As previously mentioned, Nrf2 has been shown to repress pro-inflammatory cytokine gene transcription [58] and conversely to increase the anti-inflammatory activity. This has been confirmed by the observation that Nrf2-deficient mice displayed increased pulmonary inflammation following LPS treatment and that Nrf2 inducers significantly attenuated this effect [59]. Similarly, Nrf2-deficient mice resulted to be more susceptible to the effects of different carcinogens and developed much more extensive tumours compared with those developed by the control wild-type mice [60]. To corroborate the preventive and anti-tumour function of Nrf2, many natural compounds such as resveratrol or curcumin that are well known for their anti-tumour properties, seem to exert chemopreventive action through the expression of Nrf2-induced genes [61]. It is thus not surprising that different compounds, including both natural substances and synthetic chemicals, acting by promoting Nrf2 preventive activity are currently considered as promising anti-tumour agents and some of them are already used in clinical trials for different types of cancer. For instance, sulforaphane, a well-known Nrf2 activator has been widely used as both cancer preventive and anti-tumour agent [62,63]. Trans stilbenes such as resveratrol have been shown to activate Nrf-2 and exert potent anti-tumour action inhibiting cancer invasion and metastasis [64,65].

The anticancer properties of curcumin, another Nrf2 activator, have been widely described and curcumin combination cancer therapy is under evaluation

The other side of the coin is that Nrf2, when overexpressed or constitutively activated may promote cell transformation and increase tumour resistance mechanisms as discussed below.

### 3.2. The Pro-Tumour Role of Nrf2

While many studies indicate that Nrf2 prevents cell stress in normal cells and inhibits tumourigenesis, a substantial amount of data support a pro-tumour role of Nrf2 since many types of tumours display Nrf2 high levels or overstimulation of Nrf2-mediated signalling.

Mutations in the Nrf2 gene in the Keap1 interaction domain, resulting in loss of Keap1 binding and consequent increase of Nrf2-mediated response, were found in several tumours including non-small cell lung cancer (NSCLC), hepatocarcinoma (HCC), multiple myeloma (MM), head and neck carcinoma (HNC), oesophagal carcinoma (ESC), bladder cancer (BC) [66,67].

Mutations can also occur in the principal Nrf2 repressor, Keap1 or component of the E3 ubiquitin-ligase complex Cullin 3 (CUL3), with loss of function that renders these factors unable to bind Nrf2 and thus result in persistent Nrf2 activation [68].

Besides somatic mutations, Nrf2 activation in cancers can occur by other means. Overexpression of Nrf2 has been recently found in acute myeloid leukaemia (AML) and is associated with genomic instability, gene mutation burden and drug resistance [69]. Although mutations in the Nrf2 gene have not been characterised in brain tumours [70], Nrf2 was found to be hyperactivated in a cohort of glioma patients together with p62 upregulation [71]. More recently, high Nrf2 expression was found in glioma tissues compared with non-glioma specimens and the natural compound corilagin was shown to downregulate Nrf2 expression and to induce apoptosis in glioma models [72]. Nrf2 was also upregulated in melanoma enhancing tumour malignancy by blocking differentiation and inducing COX2 expression [73] and promoting EGFR mediated oncogenic signalling [74]. Alternative splicing mechanisms in the Nrf2 transcript may also contribute to loss of the Keap1 binding domain and Nrf2 hyperactivation. In addition, overexpression of other Keap1 partners that compete with Nrf2 binding, such as p62 provokes Nrf2 stabilization [6].

Epigenetic changes amplifying Nrf2 amount or DNA methylation reducing Keap1 levels have been also described as further mechanisms to promote persistent activation of Nrf2 signalling [75]. In addition, a very recent paper by Walters et al. provides evidence that SUMO modification of Nrf2 regulates its nucleocytoplasmic localization, stability and transcriptional activity [76].

For a comprehensive analysis of Nrf2 signalling dysregulation in cancer and somatic mutations affecting Nrf2 and/or Keap1 in specific tumours, the review by Robertson et al. provides a wide and exhaustive description [77].

One explanation for the anti-tumour role of Nrf2 relies on its antioxidant capacity, which renders tumour cells less sensitive to drugs that induce oxidative stress overcoming the typically high ROS threshold of tumours. Accordingly, many lines of evidence indicate that Nrf2 overactivity is implicated in chemoresistance to different conventional drugs including cisplatin [78], 5-fluorouracil [79], doxorubicin [80], vincristine [81], etoposide [82] and mitoxantrone [83].

In this regard, a burst of recent literature has focused on compounds, many of which are natural, that are capable of inhibiting/downregulating Nrf2, thus overcoming chemoresistance [81,82,83,84,85,86,87].

In addition, evidence has been provided that Nrf2 silencing can sensitize colon cancer models to cell death induced by anti-tumour agents [88]. Another reason why Nrf2 can serve an oncogenic function is related to the promotion of HO-1 expression. As previously mentioned, the role of HO-1 in cancer is quite controversial since this enzyme can behave either as an anti-tumour factor or as a pro-tumour one [56,57,89]. HO-1 hyperactivation has been widely associated with tumour progression and acquired resistance to conventional chemotherapy [90,91,92]. Intriguingly, HO-1 cellular localization seems to drive different functions. Canonical HO-1 localises in the endoplasmic reticulum (ER) but the enzyme has been found to localise in other subcellular compartments including mitochondria, vacuoles, nucleus and plasma membrane. In oxidant conditions, HO-1 can be truncated (t-HO-1) and in this form can translocate to the nucleus where it exerts non-canonical functions [93]. t-HO-1 has been shown to induce Nrf2 expression, to bind to it favouring nuclear translocation, thus establishing an amplification loop. t-HO-1 can also contribute to Nrf2 stabilization into the nuclear compartment, preventing its nuclear proteasomal degradation. Moreover, t-HO-1 seems to participate in Nrf2-mediated antioxidant response and thus potentiates Nrf2-driven oncogenic function. It seems that this form is more abundant in tumour cells than in normal counterparts and that nuclear t-HO-1 correlates with clinical pathological features such as tumour grade and patient survival time [93]. In accordance with these observations, our previous studies demonstrated the existence of an Nrf2/HO-1 axis in ethanol stimulated colon cancer cells [94]. In these circumstances, the activation of NrF2 and tHO-1 at the nuclear level is actively used by cancer cells as a protective system against oxidative and ER stress to sustain cell survival and the acquisition of a more aggressive tumour phenotype.

It is worth mentioning the particular role of Nfr2 in lung cancer. Evidence has been provided that constitutive Nrf2 activation favours lung cancer development and promotes chemo-resistance and radio-resistance. Lung cancer cells with persistent Nrf2 activation seem to develop a sort of “Nrf2 addiction” and show malignant phenotypes leading to a poor prognosis in lung cancer patients [95]. Among the molecular mechanisms implicated in Nrf2-mediated malignancy acquisition in this tumour, stabilization of Bach1, a pro-metastatic transcription factor, has been described. Bach1 stabilization has been correlated with Nrf2-promoted HO-1 expression [96]. This event results in Bach1-mediated transcriptional activation of genes encoding enzymes implicated in glycolysis and lactate secretion, thereby promoting glycolysis-dependent metastasis in lung cancer cells [97]. Another key metabolic element in lung cancer is that Nrf2 controls the expression of genes encoding serine/glycine biosynthesis enzymes to support nucleotide production and tumour cell growth. Expression of these genes has been shown to confer poor prognosis in human non-small cell lung cancer (NSCLC) [98].

The pro-oncogenic function of Nrf2 signalling can be also due to its ability to modulate metabolic processes such as glucose metabolism, NADPH production, glutaminolysis, lipid and amino acid metabolism, which are somehow modified and utilized by cancer cells to guarantee proliferation and cell survival. A very nice and complete description in this regard has been provided by Lee [99] and De Blasi [19].

**Figure 5:**
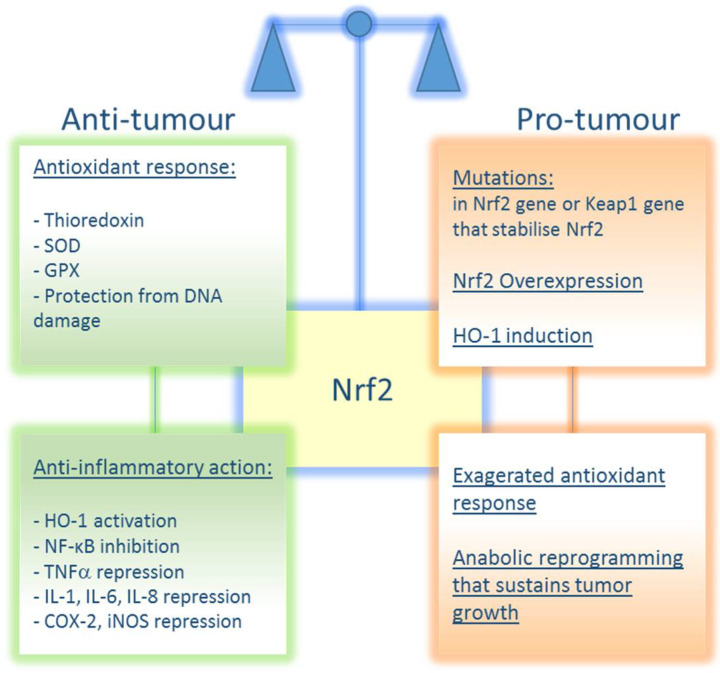
The balance between anti-tumour and pro-tumour roles of Nrf2. Under healthy conditions, Nrf2 promotes concerted antioxidant responses that protect the cells from injury and/or DNA damage. Nrf2 also promotes an anti-inflammatory action that counteracts tumour transformation. On the other hand, when Nrf2 signalling is exacerbated by Nrf2 activating mutations and/or Keap1 affecting ones, an exaggerated antioxidant response may be responsible for tumourigenesis and chemoresistance and unbalanced metabolic reprogramming may sustain cell proliferation and tumour growth.

It follows from the above considerations that Nrf2 function in cancer strictly depends on its expression level and status, whether wild type or mutated, on network alterations that render the factor constitutively active. Overall, it is possible to assert that the Janus role of Nrf2 in cancer is context dependent and not entirely controversial. A schematic representation of Nrf2 roles in cancer is reported in Figure 5.

## 4. Nrf2 Activation as a Strategy for COVID-19 Treatment

The involvement of Nrf2 signalling in coronavirus disease 19 (COVID-19) is sustained by the marked cytoprotective ability of Nrf-2 in responding to stressors and its potential role in the treatment of pathologies characterized by oxidative and/or inflammatory events. This paragraph, thus, describes the principle molecular events explaining Nrf2 antiviral action and new therapeutic perspectives in an attempt to attenuate severe acute respiratory syndrome coronavirus-2 (SARS-CoV-2) virulence by activating Nrf2 signalling.

### 4.1. The Pandemic COVID-19 and Molecular Basis of SARS-CoV-2 Infection

The pandemic COVID-19 caused by the severe acute respiratory syndrome coronavirus-2 (SARS-CoV-2) virus has dramatically affected human life and the world economy with more than 178 million confirmed cases and 3.8 million deaths (June 2021, WHO COVID-19 dashboard). At the same time, it has launched a challenge to researchers all over the world to identify the molecular features of viral infection and propagation in order to find the most appropriate therapy [100,101,102,103,104]. The SARS-CoV-2 virus is characterized by high infectivity and mutability, lethality and variant morbidity [105]. The associated disease is multifactorial due to the complex interactive network between the virus and the cellular environment that determines the great variability of symptoms [106]. There are completely asymptomatic infected subjects, while many others develop different degrees of pulmonary damages [107]. Symptoms may be mild if the infection occurs in the upper respiratory tract and gradually become severe if damage occurs at the level of alveolar cells leading to acute respiratory distress syndrome (ARDS), which is characterized by a high inflammation degree [108,109].

The pathological process starts with the entry of virus particles in the upper respiratory tract epithelial cells via angiotensin converting enzyme 2 (ACE2) [110]. This receptor, together with the transmembrane serine protease 2 (TMPRSS2), represents the root for the consequent events leading to virus infection and diffusion [111]. However, the observation that SARS-CoV-2 also infects organs with low ACE2 expression and that ACE2 downregulation occurs in a second phase of the infection makes it probable that other alternative receptors represent the virus docking sites [112,113,114].

Following infection and viral replication, new viral particles are released by the infected cells and new organs become susceptible to infection. At the same time, ROS overproduction with consequent oxidative stress occurs due to the activation of the NADPH oxidase (NOX) family [2]. In particular, NOX4 is up-regulated in lung epithelial cells after viral infections [115,116]. As we previously discussed, the increase in ROS levels is the main activating stimulus for NF-κB, which, in turn, induces the production of pro-inflammatory cytokines, provoking a high level of inflammation and stimulating lung cell death, most likely by necroptosis or pyroptosis [117,118,119].

Now it is clear that the inflammatory response of the host is crucial for the disease outcome. Severe patients suffer from symptoms correlated with lung infection but also other organs may be involved including gastrointestinal apparatus, central nervous system, heart and liver [120]. At the lung level, the infection is associated with the recruitment of monocytes differentiating into pro-inflammatory macrophages (M1), which produce a large amount of pro-inflammatory cytokines such as interleukins (IL1, IL6, IL18) and chemokines. In healthy individuals, these events facilitate the recovery, which is associated with the switch from M1 to M2 anti-inflammatory phenotype of macrophages population. On the other hand, when the SARS-CoV-2 virus infects immunocompromised subjects or with other co-morbidity of metabolic type, the inflammatory reaction becomes much more severe leading to what is generally indicated as “cytokine storm”, prevalently induced by M1 macrophages [121,122]. Very high levels of IL1, TNF-1α and IL6 are present in COVID-19 patients and are responsible for all the changes that lead to tissue fibrosis and damage in both the lung and many other organs. The cytokine storm and the oxidative stress condition induced by increased cellular ROS levels cooperate in triggering cell death [123,124].

It has been estimated that the cytokine storm is responsible for the vast majority of COVID-19 deaths [125], therefore elucidating the molecular aspects of such a complex event may help to avoid those complications that may result fatally. Molecular research has recently evidenced the role exerted by caspase-8 activity [126]. This protease is well known for its pro-apoptotic and anti-necroptotic role [127]. However, recent evidence indicates that, during COVID-19 infection, caspase-8 seems to favour the production of cytokines and the activation of pro-inflammatory intermediates causing inflammatory types of cell death [126]. Caspase-8, for instance, is able to directly cleave pro-IL-1β, thus activating (and not inhibiting), through signal transduction, the necroptotic pathway, which is known to culminate in cell lysis accompanied with inflammation [128]. Moreover, caspase-8 has been shown to activate pyroptosis, another type of inflammatory cell death, by processing gasdermin D, a protein able to create plasma membrane pores that induced osmotic cellular lysis and release of inflammatory molecules [129]. A few months ago, (March 2021) Zhang et al. hypothesized that the cytokine storm is originated from a pyroptotic event that occurs in infiltrating proinflammatory macrophages [130]. Moreover, in lung tissues derived from COVID-19 samples, the positivity to cleaved gasdermin D was observed and immunohistochemistry confirmed macrophages as the main site of positivity [130]. In this context, it is interesting to consider that Nrf2 has been reported to exert a significant inhibition of the inflammasome, the supramolecular complex involved in pyroptosis [131]. Whether Nrf2 may directly inhibit caspase 8 [116] remains to be elucidated even if caspase inhibition, including caspase 8, has been widely documented following Nrf2 activation [132]. The role of Nrf2 in viral diseases and specifically in COVID-19 is discussed below.

### 4.2. Nrf2 and COVID-19: Molecular Aspects

Made these premises, the possible relationship between the severity of COVID-19 infection and Nrf2 expression is easily predictable based on two considerations:

1. The widely described dysregulation of Nrf2 signalling in viral infections, including respiratory ones and COVID-19 in particular.
2. The observation that co-morbidities or risk factors that predispose to the most serious forms of COVID-19 are strictly associated with Nrf2 reduced expression.

Concerning the first point, it is well known that during viral infection the increase in ROS production and the consequent oxidative stress is crucial for the viral replication as demonstrated following influenza virus [115], hepatitis C (HCV) [133], hepatitis B (HBV) [134] and human immunodeficiency virus (HIV) [135] infections. Activation of oxidative stress by different types of virus occurs despite the specific viral genome (DNA or RNA). For instance, Lee nicely described the modulation of virus-induced oxidative stress via the Nrf2-dependent antioxidant response, making different examples of specific viral types [136].

As a consequence, infected cells respond to this imbalance by activating antioxidant response, which is mainly mediated by the Nrf2 pathway and accompanied by the expression of a plethora of antioxidant genes [137] as discussed before.

It is therefore not surprising that viruses tend to block the expression of Nrf2 as observed in the respiratory syncytial virus or HCV infection [138]. However, an opposite situation has been also described. An example is represented by HIV Tat protein that can simultaneously induce ROS production and the Nrf2 pathway. Nevertheless, if the infection is prevalent, it is reasonable to hypothesize that the response triggered by Nrf2 signalling is too weak or delayed [139]. The outcome of Nrf2 activation has been also related to the specific phase of virus infection. During HCV infection, for instance, in the acute phase, the activation of the Nrf2 pathway defends infected cells from inflammatory response, while during the chronic phase its inhibition restores the oxidative imbalance, thus favouring the virus persistence [140].

Specifically, regarding COVID-19, its very recent origin together with the difficulty in creating experimental animal models, make it arduous to identify molecules and therapeutic strategies. However, in analogy to other viral infections, the hypothesis of Nrf2 involvement was immediate. Data from lung biopsies from COVID-19 patients evidenced a marked reduction in the expression of genes of Nrf2 mediated antioxidant response [141]. In addition, the same authors showed a decrease in the levels of HO-1 and NADPH oxidoreductase 1 in Vero cells infected by SARS-CoV-2 [141].

Concerning the second point, indirect indications for the involvement of Nrf2 in COVID-19 are related to the observation that Nrf2 reduced expression occurs when co-morbidities or increased risk factors determine the most serious forms of COVID-19. Notoriously, the main risk factors for severe forms of COVID-19 are age, obesity, hyperglycaemia, sex (being males more susceptible than females to the disease). It is intriguing to observe that all these conditions are accompanied by reduced levels of Nrf2. Ageing is associated with an imbalance between ROS production and antioxidant defences. Accumulating data indicate a reduced efficiency of Nrf2 antioxidant pathways in ageing. Nrf2 signalling changes can be due to increased Nrf2 degradation, blockage of its nuclear translocation, changes in post-transcriptional modifications, and an increase in specific regulatory miRNAs [142].

Similar considerations can be made about hyperglycaemia. This dysmetabolism is also associated with oxidative stress. A recent study has demonstrated that the inhibition of Nrf2 signalling could significantly promote the incidence of type I diabetes mellitus, and, on the other hand, its reactivation reduces oxidative stress in pancreatic β-cells [143]. At the same time, the absence of Nrf2 protects from insulin resistance in long-term high-fat diet feeding by decreasing adipose tissue inflammation [144,145].

From a molecular point of view, a number of pathways and molecules resulted to be modified during SARS-CoV-2 infection, many of which are closely related to the reduction in Nrf2 levels.

It is possible to speculate that in the first phase of the infection Nrf2 is activated as a cellular response, whereas following virus propagation a decrease in Nrf2 occurs.

As a response to viral infection, cells activate ER stress and the associated UPR. This event leads to the activation of one of the UPR mediators, PERK which phosphorylates and activates Nrf2 [146]. Moreover, the ER stress-induced activation of protein kinase R upregulates p62 that, as previously mentioned, is able to compete with Keap1 for Nrf2 binding, to stabilise Nrf2 [147].

It is thus clear that Nrf2 represents a defence factor against the virus. In particular, evidence has been provided that active Nrf2 inhibits the transcription of angiotensin converting enzyme 2 (ACE2) which, interacting with the spike protein of the virus capsid, represents the site for virus entry [148]. Notably, overexpression of ACE2 is recurrent in subjects with cardiovascular diseases who, assuming ACE inhibitors, show an imbalance in the ACE/ACE2 ratio. For this reason, these patients have a major risk to evolve a more severe form of COVID-19 [149].

Another important protective action of Nrf2 during SARS-CoV-2 infection is related to its ability to inhibit STING (stimulator of interferon genes), by reducing STING mRNA stability. STING is a factor that regulates the transcription of interferons (IFNs) and is related to host cell defence. As described by Olagnier et al., an interesting relationship between metabolic reprogramming and antiviral cytosolic DNA sensing in human cells involves Nrf2 [150].

However, IFNs exert a double role during virus infections since they can inhibit virus replication in the initial phase of the infection but with increasing concentrations, they sustain pulmonary infiltration of macrophages. Moreover, an inverse relationship exists between Nrf2 and type I IFNs. Lei et al. have demonstrated, indeed, that IFNs are able to suppress the activation of Nrf2, thus increasing oxidative stress, pro-inflammatory cytokine responses, and metabolic dysfunction [151]. It is thus predictable that Nrf2 becomes inactivated when infection proceeds.

The protective role of Nrf2 against SARS-CoV-2 is also sustained by its role in counteracting cytokine storms. This action has been documented through inhibition of the production of IL6, one of the principal pro-inflammatory cytokines [152].

In addition, as above reported, antioxidant Nrf2 signalling and pro-inflammatory NF-κB-mediated pathway are strictly related to each other. Their interplay is also involved in COVID-19. A cytokine storm is strongly sustained by NF-κB pathway, whereas Nrf2, through NF-κB inhibition, promotes a defence function with the consequent reduction of the inflammatory burst. The intensity of ROS production may be responsible for the control of the NF-κB/Nrf2 balance. A dramatic ROS production sustains NF-κB pathway, which is accompanied by Nrf2 inactivation, resulting in an amplification of the inflammatory loop. Conversely, a pre-condition of antioxidant Nrf2-dependent redox status or an efficacious recovery of the Nrf2 pathway protects the cells from severe compromising effects [153].

Furthermore, the modulation of NF-κB/Nrf2 interaction and the reduced IL-6 and IL-1β expression by ozone therapy seem to have an impact on the cytoprotection and blockage of viral replication, thus being potentially useful in SARS-CoV-2 disease [154].

Another interesting protective effect of Nrf2 is due to the upregulation of HO-1, which is fundamental for cell defence against the coronavirus. Many indications sustain, indeed, that products of heme degradation determined by HO-1 (biliverdin, CO and Fe^2+^) display anti-SARS-CoV-2 potential, similar to that observed during other viral infections [155,156]. The aforementioned evidence and considerations suggest a potential role of Nrf2 in preventing/counteracting SARS-CoV-2 infection and propagation.

### 4.3. Can Nrf2 Activators Display Anti-COVID-19 Potential?

Considering what was previously discussed, a hypothesis arises that nutrients or synthetic molecules with documented Nrf2 activating activity may counteract COVID-19. In this regard, a relationship between the diet followed in different countries and COVID-19 death rates has been reported together with the effects of dysmetabolic conditions on the disease gravity (obesity, Type 2 diabetes insulin resistance). In particular, the authors demonstrated that the main Nrf2-interacting nutrients/natural compounds have positive effects on diseases associated with oxidative stress [157]. Consequently, attenuating the redox imbalance by Nrf2-activating nutrients could result in a less severe form of COVID-19. The list of foods containing such nutrients or bioactive compounds is quite long. Actually, the list includes vegetables, fruits and fermented foods which are known for their anti-inflammatory and anti-oxidant abilities, that are associated with specific classes of molecules such as flavonoids, and many others [157,158]. Although studies on the real effectiveness of these molecules in protecting against COVID-19 are ongoing, the concept that a correct diet, enriched with foods having Nrf2-mediated antioxidant capacity, could be valid since it may predispose people to develop an eventual infection with mild symptoms.

Nrf2 can be also activated in other ways. Recently, the possible beneficial outcomes of COVID-19 pneumonia by activating the Nrf2 pathway via chemical and ionizing radiation were analysed by Calabrese et al. [159], while Martínez-Sánchez et al. verified the potential of ozone therapy [154].

Apart from specific nutrients, many other molecules with the Nrf2-activating effect have been well described. The methyl ester of fumaric acid (dimethylfumarate, DMF), which is already in use for the treatment of psoriasis and multiple sclerosis, is a potent activator of Nrf2. Since both these diseases share with COVID-19 an inflammatory basis, this drug has been indicated as a potential therapeutic candidate [160,161]. From a molecular point of view, DMF has been shown to inhibit virus entry by inducing anti-protease protein SPLI expression and inhibiting transmembrane serine protease TRMPSS2, a cell surface receptor, required for SARS-CoV-2 entry to target host cells [162]. DMF also inhibits the upregulation of ACE2 and anti-viral mediators such as retinoic acid-inducible gene I (RIG-I) and interferons (INFs). Induction of Nrf2-mediated anti-oxidant response promoted by DMF also inhibited NF-κB pathway, thus contributing to attenuating inflammation.

Bardoxolone methyl is a pentacyclic triterpenoid with anti-inflammatory activity and a potent activator of Nrf2. It is currently in clinical trials against hyperglycaemia and has been recently suggested against COVID-19, being able to inhibit SARS-CoV-2 replication [163]. However, its detrimental effects on microvascular endothelium represent a limitation of its employment [164].

Another Nrf2 activator is 4-octyl itaconate, an analogous of itaconate with immunoregulatory and antioxidative effects produced by decarboxylating cis-aconitate. This compound seems quite promising since it showed suppression of inflammatory response in COVID-19 [161]. Other potential drugs are sulforaphane, isolated from broccoli, and its encapsulated variant sulfodarex with demonstrated anti-inflammatory response in lungs, acting as NADPH:quinone oxidoreductase 1 inducer [165].

**Figure 6:**
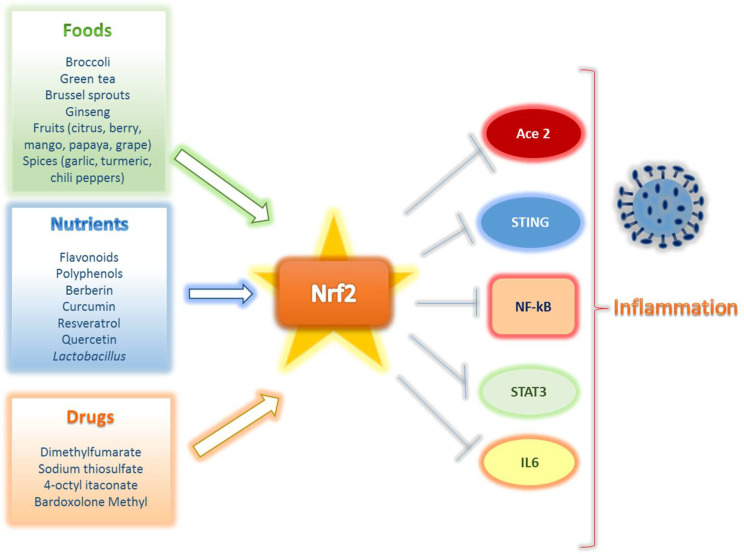
The potential of Nrf2 activators in combating COVID-19. Foods containing antioxidants that stimulate Nrf2, such as vegetables; specific natural compounds; synthetic drugs or metabolites can produce Nrf2 activation thus targeting those factors that are involved in SARS-CoV-2-induced inflammation and consequent cytokine storm.

Notably, it is worth considering that many of these molecules act on several fronts; many of them together with Nrf2-activating action are also able to inhibit NF-κB or STAT3/5 [166], thus contributing to reducing inflammation (Figure 6). This double action on Nrf2-mediated cytoprotection and NF-κB-mediated anti-inflammatory effects, make these molecules very promising candidates in COVID-19 treatment.

## 5. Conclusions

The diversified roles of Nrf2, ranging from antioxidant to metabolic and anti-inflammatory, render the factor a cell fate determinant and a key player in those mechanisms regulating cell transformation and response to viral infections. Given the molecular complexity that characterises cancer development and progression, it is not easy to classify a factor like Nrf2 as positive or negative in the disease outcome. If we can consider Nrf2 a protective factor when it prevents oxidative stress and inflammation associated with tumourigenesis, we have to consider it an oncogenic factor when it is hyper-stimulated or overexpressed, thus causing the opposite effects. Distinguishing such behaviour in different situations appears important to understand the molecular basis of cancer and to perform appropriate anti-tumour targeted therapies either stimulating or inhibiting Nrf2.

If the factor exerts a double role in cancer, in the context of viral infection it seems to display a preventive and protective action. This is mainly associated with the significant anti-inflammatory Nrf2 function that prevents disease complications. Possible beneficial outcomes of COVID-19 are thus associated with activating the Nrf2 pathway, rendering Nrf2 activators, either of natural or synthetic origin, key substances to combat the disease.
